# Water transport control in carbon nanotube arrays

**DOI:** 10.1186/1556-276X-9-559

**Published:** 2014-10-08

**Authors:** Matteo Fasano, Eliodoro Chiavazzo, Pietro Asinari

**Affiliations:** 1 Dipartimento Energia, Politecnico di Torino, Corso Duca degli Abruzzi, 24, 10129 Torino, Italy

**Keywords:** Water diffusion, Carbon nanotubes, Nanoconfinement, Supercooled water, Carbon mats, Nanotube arrays, Membranes, Molecular sieves

## Abstract

Based on a recent scaling law of the water mobility under nanoconfined conditions, we envision novel strategies for precise modulation of water diffusion within membranes made of carbon nanotube arrays (CNAs). In a first approach, the water diffusion coefficient *D* may be tuned by finely controlling the size distribution of the pore size. In the second approach, *D* can be varied at will by means of externally induced electrostatic fields. Starting from the latter strategy, switchable molecular sieves are proposed, where membranes are properly designed with sieving and permeation features that can be dynamically activated/deactivated. Areas where a precise control of water transport properties is beneficial range from energy and environmental engineering up to nanomedicine.

## Background

Carbon nanotubes (CNTs) and other carbon-based particles are used as fillers in a large variety of composite materials because of their superior thermal, mechanical, and electrical properties [1-4].

Patterns of vertically aligned CNTs (also known as carbon nanotube arrays (CNAs)) can be immersed in polymer or ceramic matrices for obtaining nanoporous materials, which are characterized by precisely controlled pore width and density. CNAs are typically produced by either physical [5] or chemical [6,7] vapor deposition on catalyst or substrate patterns predesigned by nanolithography processes; however, alternative production approaches - such as self-assembly on biological templates [8] or DNA-mediated [9] - have also been recently proposed. Well-ordered nanoporous membranes are obtained by CNAs incorporation across polymer or ceramic matrices by spin-coating [10] or conformal encapsulation [11], respectively. Plasma-etching treatments can then open up CNT tips, whereas plasma-oxidation processes functionalize channel entrances for gatekeeping purpose [12].

CNA-based materials may find biomedical or engineering applications such as nanostructured filters, separators, detectors, or vectors with diagnostic or therapeutic function. Concerning biomedical applications, CNAs are ideal building blocks for designing artificial biomembranes capable of mimicking functionalities of Nature’s channels (e.g., aquaporins), as recently investigated both experimentally [13] and numerically [14]. These nanoporous materials are also used in devices for controlled transdermal drug delivery [15] as well as for DNA/RNA amplification [8], sensing [16], or translocation [17-19]. Engineering applications of CNAs mainly exploit their ability to selectively trap nanoimpurities (e.g., wastewater treatments [20,21]) or ions (e.g., seawater desalination [22-24]) dispersed in water, as well as their gas separation [25] and catalysis properties [26], especially for fuel cell technology. Further peculiar properties of CNTs entail an even broader applicability of CNAs in engineering: super-hydrophobicity for self-cleaning surfaces [27,28], phonon dragging by fluid flow for energy harvesting or nanosensing [29,30], and optical properties for electrical coupling in photonic [31] or photothermal devices [8].

Although mechanical, thermal, and electrical properties of CNTs have been deeply investigated [1,32], the physical understanding of diffusion properties of fluids through their pores is still incomplete [33,34]. Transport diffusivity *D*_*T*_ of water inside narrow CNTs is order of magnitude faster than bulk one, as demonstrated by both computational calculations and experiments [12,13,35-37]. Such flow rate enhancement is due to slip flow conditions of water within CNT nanopores, which is governed by the liquid structure and collective molecular motion induced by both mechanical and electrical smoothness of CNT walls and their confinement effect on water. Predicting the water self-diffusivity (i.e., mobility) *D* under nanoconfined conditions is also of interest in several fields [38]. Computational studies agree with a progressive reduction of water mobility by decreasing the nanotube diameter [33,39]: a physical interpretation - based on supercooling properties of nanoconfined water - and quantitative prediction of the phenomenon have been recently formulated by Chiavazzo and collegues [38], whereas novel and improved experimental techniques may further support these studies in the near future [34].

The apparent discrepancy between low *D* and high *D*_*T*_ of water within narrow CNTs (i.e., subnanometer diameters) may be explained by invoking the Maxwell-Stefan (M-S) diffusion model, which allows to analyze mass transfer phenomena from a more fundamental point of view [40-45]. By resorting to *Ð*_*i*_ (M-S diffusivity, also known as corrected diffusivity) and to *Ð*_*ii*_ (self-exchange coefficient) [34], it can be easily demonstrated that:

(1) $$\frac{1}{D}=\frac{1}{{\text{Ð}}_{i}}+\frac{1}{{\text{Ð}}_{\text{ii}}}.$$

In other words, self-diffusivity *D* within a nanoporous matrix (or, analogously, self-diffusion drag resistance 1/*D*) is dictated by (a) species *i*-wall and (b) species *i*-species *i* interactions, respectively [42]. Moreover, by recalling the definition of thermodynamic factor $\Gamma \equiv \frac{\partial \ln p}{\partial \ln c}$ (*c* is the equilibrium concentration with respect to the pressure *p*) [40,46], which can be interpreted as an extra driving force for transport diffusion, transport diffusivity is found to be correlated to the M-S one as follows:

(2) $$D_{T}=\Gamma {\text{Ð}}_{i}.$$

When CNTs with subnanometer diameters are considered, single-file diffusion regime occurs, which induces a sudden increase of the drag experienced by water molecules in passing each other (i.e., 1/*Ð*_*ii*_→*∞*). Hence, due to Equation 1, 1/*D*→*∞* and *D* assumes near zero values, which means that water is totally nanoconfined within the CNT even if *D*_*T*_ may still attain quite large values [34].

Instead, when diameters larger than 1 nm are considered, Einstein-like diffusion of water is recovered. However, if CNT diameter are sized so that inner water is still predominantly under nanoconfinement conditions (i.e., wall-water interactions are significantly larger than water-water ones), 1/*Ð*_*ii*_≪1/*Ð*_*i*_[34] and Equations 1 and 2 yield *D*≊*Ð*_*i*_ thus

(3) $$D_{T}≊\mathrm{\Gamma D.}$$

In this particular regime, self- and transport diffusivities are correlated by means of the thermodynamic factor. Equation 3 (also known as Darken’s equation) was first suggested by Richard Barrer [47,48], and it has been demonstrated both theoretically and experimentally to be a reliable correlation between self- and transport diffusion under the aforementioned conditions [34,49].

For these reasons, the *a priori* prediction of self-diffusion coefficient of water within CNTs allows a more rational design of the CNA-based technologies relying on transport properties of water (e.g., mass separators, catalytic converters, selective filters, molecular sensors, nanosized chemical or biological reactors). Here, classical molecular dynamics (MD) simulations and the recent modeling of water transport in nanoconfined conditions [38] are synergistically used for a systematic prediction of self-diffusivity of water in CNAs, according to different pore width distributions and electrostatic fields. Results pave the way to a precise design of transport properties of water in CNA-based devices.

## Presentation of the hypothesis

Water mobility is progressively reduced while approaching solid surfaces at the nanoscale because of the confinement effect induced on water molecules by attractive nonbonded interactions at the solid-liquid interface [50]. The reduction in water mobility implies a smaller self-diffusion coefficient *D*, ranging from bulk values to almost null ones according to the nanoconfinement conditions, depending on the geometric, chemical, and physical factors of a given configuration. Peculiar thermodynamic properties of supercooled water have a key role in interpreting the reduction of *D* in nanoconfined environments [51].

Following the approach in reference [38], if isothermal conditions are considered, the intensity of water nanoconfinement scales with a dimensionless parameter $\theta =\frac{V_{\text{in}}}{V_{w}}$, being *V*_in_ the volume where a solid surface exerts a non-negligible influence on the water dynamics and *V*_*w*_ the overall volume occupied by water in the considered configuration. *V*_in_ can be estimated by introducing the characteristic length of nanoconfinement *δ*, which is defined as the distance where thermal agitation of water is still significantly influenced by van der Waals *U*_vdw_ and Coulomb *U*_c_ solid-liquid interactions, namely where kinetic energy of water *k*_*B*_*T* equals solid-liquid effective potential *U*_eff_=*U*_vdw_+*U*_c_. Note that water molecules beyond the characteristic length *δ* progressively tend to behave as bulk ones because they escape the potential well generated by the solid wall, which is subject to a power decrease along the direction normal to the surface.

**Figure 1:**
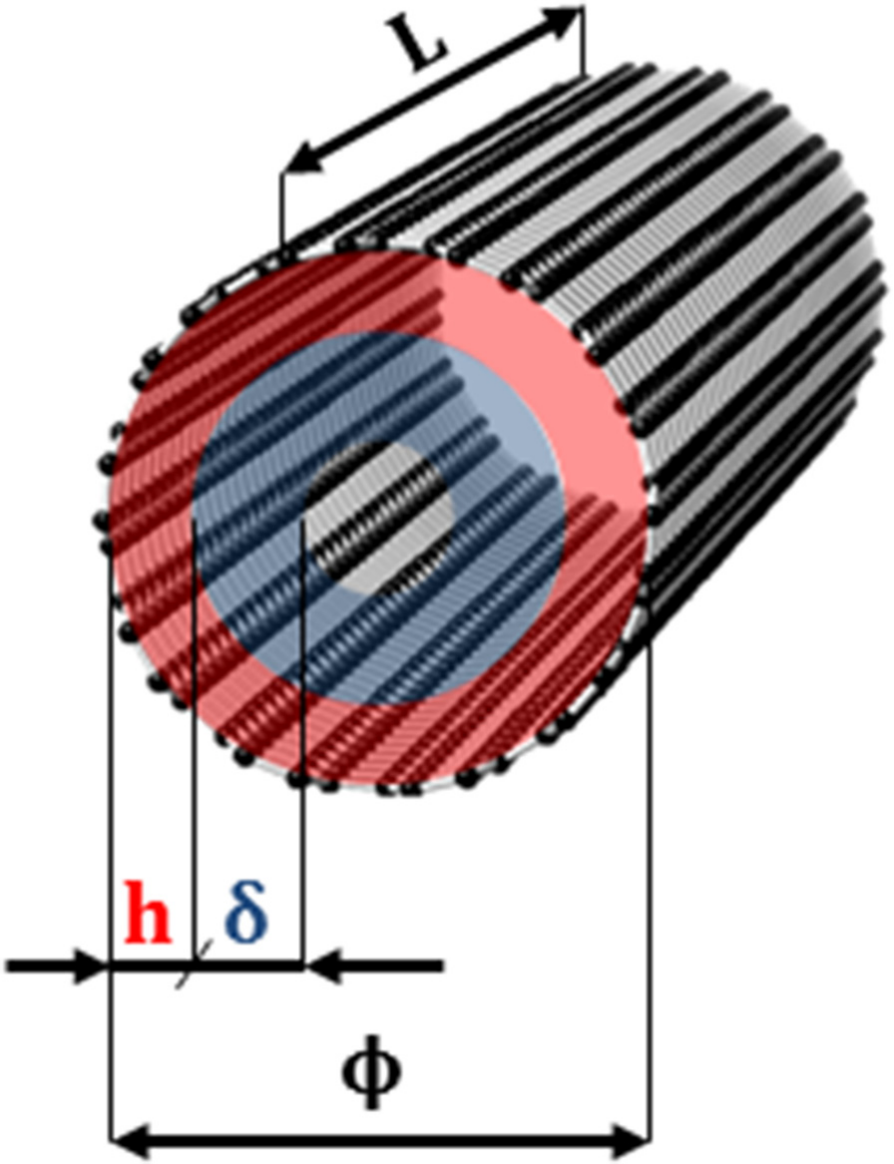
**CNT geometry.** The geometric quantities of CNT, needed for evaluating the scaling parameter *θ* in Equation 4, are schematically shown.

In case of CNTs immersed in homogeneous impermeable polymer or ceramic matrices, water only interacts with the inner surface of nanotubes. Hence, *θ* can be more precisely reformulated as:

(4) $$\theta =\frac{\frac{\mathrm{\pi L}}{4}\left[\phi_{e}^{2}-{\left(\phi_{e}-2\delta \right)}^{2}\right]}{\frac{\mathrm{\pi L}}{4}\phi_{e}^{2}}=\frac{4\phi_{e}\delta -4\delta^{2}}{\phi_{e}^{2}},$$

being (see Figure 1) *L* the nanotube length; *ϕ*_*e*_=*ϕ*-2*h* the solvent accessible diameter of the nanotube, where *h*≈0.34 nm is the minimum distance of approach between carbon atoms and water [52] and $\phi =\frac{a}{\pi }\sqrt{\left(n^{2}+\text{nm}+m^{2}\right)}$ the nominal diameter of a nanotube, as from its chirality (*n*,*m*) with *a*=0.246 nm. Note that when $\delta \geq \frac{\phi_{e}}{2}$ water is considered as totally confined, thus *θ*=1. By considering a CNA made out of *N* CNTs, the average value of the scaling parameter $\bar{\theta }$ in the composite material can be then defined as:

(5) $$\bar{\theta }=\frac{\overset{N}{\underset{i=1}{\sum }}4\phi_{e,i}\delta_{i}-4\delta_{i}^{2}}{\overset{N}{\underset{i=1}{\sum }}\phi_{e,i}^{2}}.$$

The quantity *δ* can be computed by a well-defined algorithm [38] once nanoconfined geometry, Connolly surface of solid [53] and solid-water nonbonded interaction potentials are given. Connolly surfaces are generated from MD trajectories, which are produced by the following procedure: (i) CNT structures are solvated in triclinic boxes of water (SPC/E model [54]) with different volumes and periodic boundary conditions along *xyz* directions, (ii) the system made by nanotube and water is energy minimized and equilibrated, (iii) a molecular dynamics simulation of the whole setup is then performed up to 200 ps in canonical ensemble, by applying a temperature coupling on the system (time constant = 0.1 ns, *T*= 300 K) [55]. Two types of atomic interactions are taken into account in the simulations: (i) bonded interactions, modeled as harmonic stretching and angle potentials and (ii) nonbonded interactions, accounting for Van der Waals and electrostatic forces, modeled as a 12-6 Lennard-Jones potential. Further details on the employed force field are reported elsewhere [38,52,56,57]. Simulations are carried out with a leapfrog algorithm (1 fs time step) by means of GROMACS software. [58] Nanotubes with different size (*ϕ*,*L*) or partial charge (*q*_*C*,*i*_) on carbon atoms are simulated, in order to assess geometric and electrostatic effects on *δ*.

Following reference [38], once *θ* is computed by Equation 4, *D* can be readily predicted as:

(6) $$D\left(\theta \right)=D_{B}\left[1+\left(\frac{D_{C}}{D_{B}}-1\right)\theta \right],$$

where *D*_B_ and *D*_C_ are self-diffusion coefficients of bulk (2.60×10^-9^ m^2^ s^-1^ at 300 K for SPC/E water model [59]) and confined water, respectively. Equation 6 can be simplified by safely assuming $\frac{D_{C}}{D_{B}}\approx 0$[38] and thus

(7) $$D\left(\theta \right)\approx D_{B}\left(1-\theta \right),$$

namely a linear decrease of water mobility with the scaling parameter *θ*. In other words, since Equation 4 can be approximated by $\theta \approx \frac{4\phi_{e}\delta }{\phi_{e}^{2}}$ because usually *ϕ*_*e*_≫*δ* thus $\frac{\delta^{2}}{\phi_{e}}\approx 0$, Equation 7 yields:

(8) $$\frac{D}{D_{B}}\approx \left(1-\frac{4\delta }{\phi_{e}}\right),$$

which is valid for $\frac{4\delta }{\phi_{e}}\leq 1$.

Inspection of Equation 8 reveals that strategies aiming at tailoring the self-diffusivity of water within CNTs can be based on the design of either the tube diameter or the confinement potential (i.e., CNT-water interactions, by introducing defects, functionalizations, or external electrostatic fields).

## Testing of the hypothesis

Implications of Equation 8 on CNA-based technologies are analyzed by means of MD simulations, which are here used for estimating *δ* of CNTs in the considered setups. In particular, effects of CNT geometry or electrostatic field on *D* are explored, in order to suggest experimental guidelines to precisely control water self-diffusion in CNAs under static conditions.

First, different width distributions of CNA pores are considered. Characteristic length of nanoconfinement *δ* of CNTs are then calculated with different *ϕ* (i.e., from 0.8 to 14.0 nm) and *L* (i.e., from 5 to 50 nm) by MD simulations. Results show that nanotube geometry has no significant effect on the characteristic length of nanoconfinement, being approximately around *δ*=0.37 nm.

Current experimental techniques allow to produce arrays of nanotubes with size, pattern, and areal density precisely defined, by simply adjusting production procedure or parameters [6,60,61]. While CNT length usually spans from tens to thousands of nanometers, CNT diameter can reach few nanometers width or even less [11,62]. However, CNAs include pores with a Gaussian distribution of diameters *ϕ*, where significant standard deviations *σ* of the average value *μ* can be encountered because of the lack in repeatability and quality of current production techniques [11,13,62].

**Figure 2:**
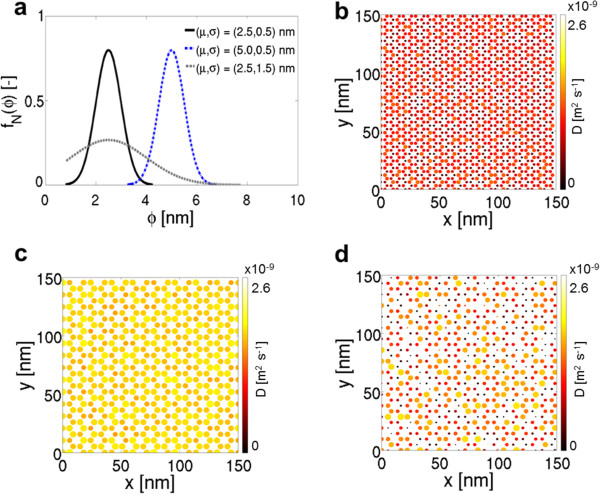
**Self-diffusivity of water in CNAs with Gaussian distribution of pore width.(a)** Three Gaussian distributions of CNT diameters are considered, with different mean values *μ* or standard deviations *σ*. **(b)** Self-diffusion coefficient of water nanoconfined within CNTs distributed with Gaussian frequencies *f*_*N*_ and *μ*= 2.5 nm, *σ*= 0.5 nm; **(c)***μ*= 5.0 nm, *σ*= 0.5 nm; **(d)***μ*= 2.5 nm, *σ*= 1.5 nm.

In Figure 2a, three Gaussian distributions of *ϕ* are considered (being *L*=100 nm fixed), according to different (*μ*,*σ*) values, namely: 2.5 nm, 0.5 nm; 5.0 nm, 0.5 nm; and 2.5 nm, 1.5 nm. Note that *ϕ*= 0.8 nm is the narrowest CNT experimentally observable, whereas a distribution interval *ϕ*∈[*μ*-2*σ*,*μ*+2*σ*] is taken into account. According to Equations 5 and 7 and the characteristic length of nanoconfinement *δ* from MD runs, overall *D* decreases as the average *ϕ* is reduced: $D\left(\bar{\theta }\right)$ is 0.99×10^-9^ m^2^ s^-1^ for *μ*= 2.5 nm (Figure 2b) whereas it increases to 1.78×10^-9^ m^2^ s^-1^ for *μ*= 5.0 nm (Figure 2c). Moreover, standard deviation has also a significant role in tailoring local and average *D* of water within CNAs. For example, while in CNT distribution represented in Figures 2b,d the mode (0.89×10^-9^ m^2^ s^-1^) and the minimum (near 0 m^2^ s^-1^) *D* values are the same, the maximum *D* shifts from 1.40×10^-9^ to 2.47×10^-9^ m^2^ s^-1^, respectively. Moreover, a *σ* increase from 0.5 to 1.5 nm leads a 40% rise in $D\left(\bar{\theta }\right)$ to 1.41×10^-9^ m^2^ s^-1^.

**Figure 3:**
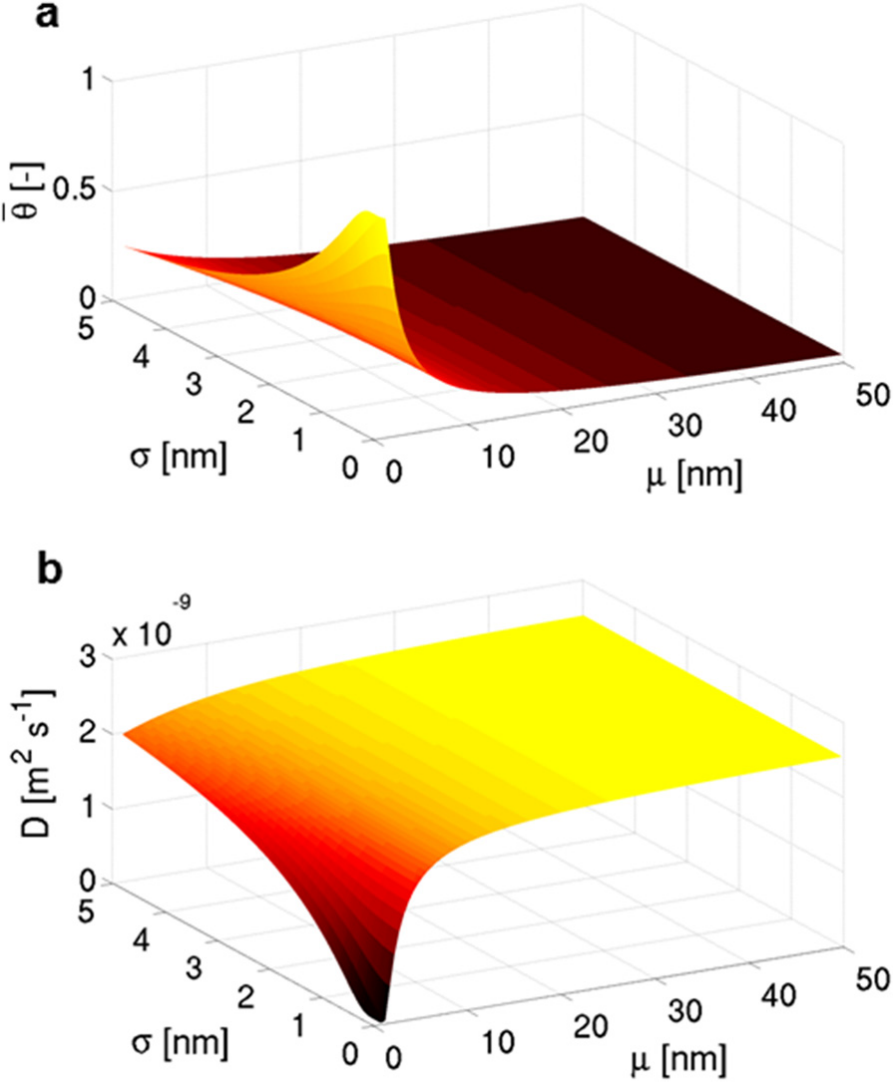
**Effect of pore diameter distribution on water nanoconfinement in CNA. (a)** CNAs with Gaussian distribution of pore width are systematically analyzed (*μ*∈ [0.8, 50]; *σ*∈ [0, 5]), and both the average scaling parameter $\bar{\theta }$ (i.e., the mean magnitude of water nanoconfinement in a CNA section) and **(b)** the overall self-diffusivity of entrapped water $D\left(\bar{\theta }\right)$ (Equation 7) are evaluated.

A more systematic analysis of the influence of *μ* and *σ* on $\bar{\theta }$ (thus on the overall *D*) is then performed. Results in Figure 3 show a synergistic contribution of *μ* and *σ* in the reduction of *D*. In fact, both large *σ* values and large *μ* values imply *D*≈*D*_B_ (regardless of other quantities); whereas if both average and standard deviation of CNT diameter distributions show lower values, an exponential reduction in *D* is experienced. In other words, the average *D* of water within CNAs can be tuned not only by controlling the average pore size (i.e., *μ*) but also by the accuracy (i.e. *σ*) of the CNA production, which strongly affects the overall CNA transport properties. Note that a significant reduction (e.g., more than 25%) in overall *D* from bulk values is only achievable when CNT diameter distributions are characterized by *μ*< 7.0 nm and *σ*< 3.0 nm. Moreover, Figure 3 highlights that an average bulk behavior of water within CNA-based composite materials is expected for *ϕ*> 10 nm, independently from the size distribution; however, as already noticed in Figure 2d, highly dispersed size distributions (i.e., high *σ*) imply low local *D*, which may be of interest for size-dependent molecular sieving applications.

Second, *δ* and *D* are investigated when an electrostatic field is applied to CNA. If CNA-based composites are characterized by strong dielectric properties, the inner surface of CNTs tends to be uniformly polarized if an electrostatic field is switched on [63-65]. Therefore, MD force field is adequately modified for taking into account the charges introduced on carbon atoms (*q*_*C*,*i*_) by applying an electrostatic potential to the system.

**Figure 4:**
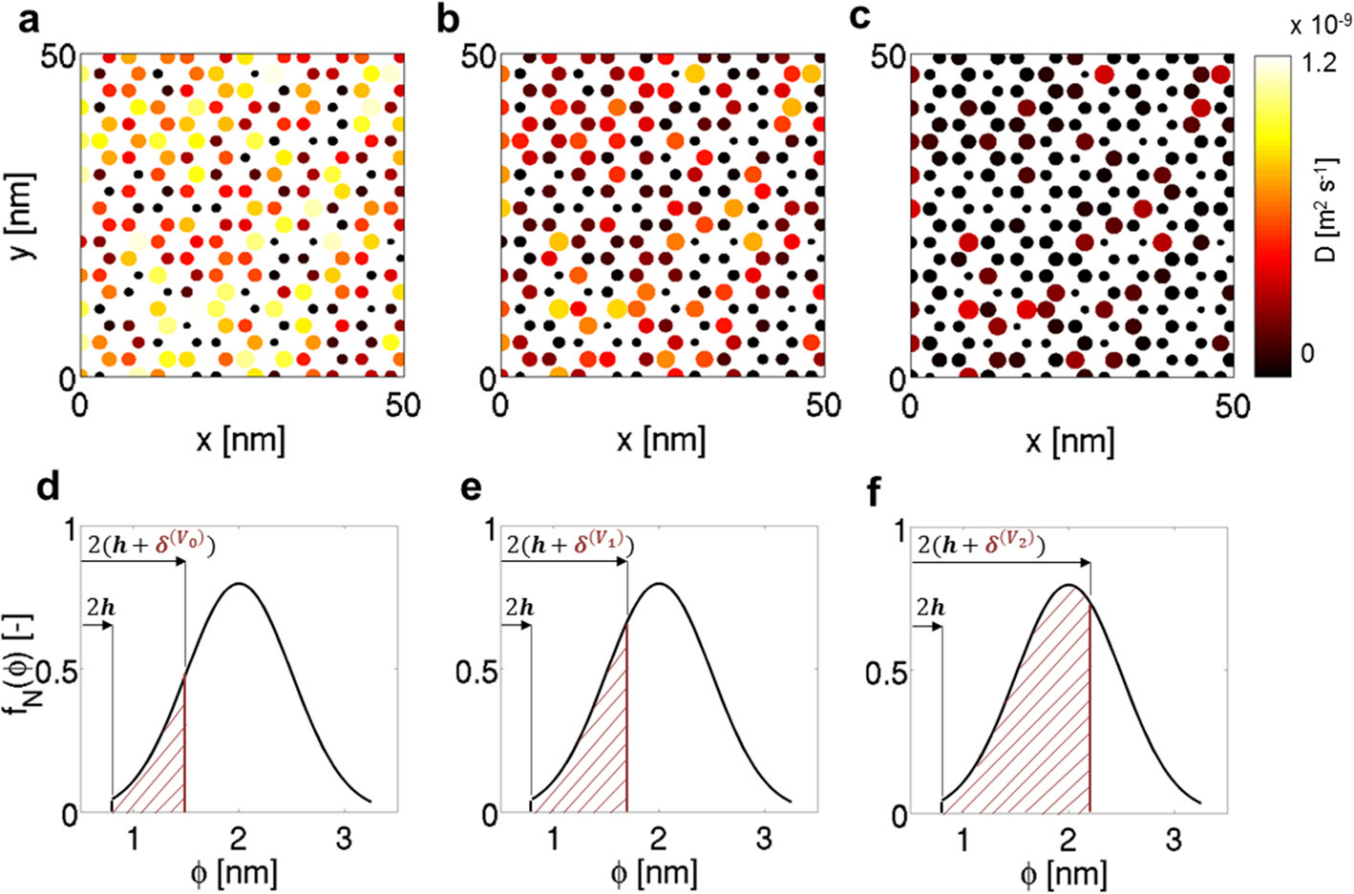
**Water nanoconfinement increase by electrostatic field application to CNA. (a)** Local *D* of nanoconfined water are plotted as predicted by Equations 4 and 6, while considering different Coulomb charges on carbon atoms for simulating the application of an electrostatic field to CNA, namely *q*_*C*,*i*_= 0 eV, **(b)***q*_*C*,*i*_= 0.5 eV or **(c)***q*_*C*,*i*_= 1.0 eV. **(d)** The considered CNA section presents pore diameters distributed with Gaussian probability (*μ*= 2.0 nm; *σ*= 0.5 nm). When no electrostatic field is applied, $\delta^{\left(V_{0}\right)}=0.37\text{nm}$ and 15% of CNA pores contain totally confined water (i.e., *θ*= 1) with near zero mobility; whereas **(e)** with *q*_*C*,*i*_= 0.5 eV, $\delta^{\left(V_{1}\right)}=0.50\text{nm}$ and the percentage rises to 32% or **(f)** with *q*_*C*,*i*_= 1.0 eV, $\delta^{\left(V_{2}\right)}=0.70\text{nm}$ and the percentage further increases to 67%. *h*≈ 0.34 nm is the minimum approaching distance between carbon atoms and water molecules.

A CNT with *ϕ*= 1.4 nm and *L*= 12 nm is chosen as a representative case, and *δ* is estimated with different *q*_*C*,*i*_. Considering a fixed CNA section (*μ*= 2 nm; *σ*= 0.5 nm), Figure 4 depicts how *D* distribution changes when *q*_*C*,*i*_= 0 eV (*δ*= 0.37 nm), 0.5 eV (*δ*= 0.50 nm), or 1.0 eV (*δ*= 0.70 nm), respectively. More specifically, $D\left(\bar{\theta }\right)=0.70\times 10^{-9}{\text{m}}^{2}{\text{s}}^{-1}$ in a neutral CNA (Figure 4a), while it drops to 0.36×10^-9^ m^2^ s^-1^ with *q*_*C*,*i*_= 0.5 eV (Figure 4b) and to 0.10×10^-9^ m^2^ s^-1^ with *q*_*C*,*i*_= 1.0 eV (Figure 4c). It is also worth mentioning that water is totally confined in 15% of CNA pores when no electrostatic field is applied (Figure 4d), whereas the percentage rises to 32% and 67% when *q*_*C*,*i*_= 0.5 (Figure 4e) or 1.0 eV (Figure 4f), respectively.

The voltage corresponding to an electrical charge *q*_*C*,*i*_ can be estimated by the classical relation: $V=\frac{Q}{C}$, where $Q=\underset{i}{\sum }q_{C,i}$ is the overall electrostatic charge on CNTs and *C* is the electrical capacitance. Experimental works show that the specific capacitance $c=\frac{C}{m}$ of pure single-walled CNTs is about 40 F g^-1^[64]; whereas it increases to 320 F g^-1^[66] or even more when CNTs are immersed in electrically conducting polymers, giving rise to ideal materials for supercapacitors. Hence, considering the previous upper and lower bounds for *c*, the voltage is found to vary within the following ranges: *V*≈ 140 to 1,195 V for *q*_*C*,*i*_= 0.5 eV and *V*≈ 280 to 2390 V for *q*_*C*,*i*_= 1 eV, which can be easily achieved by common electrostatic devices.

Few experimental techniques are currently available for validating these predicted reductions in water self-diffusion, namely by direct (e.g., diffusion nuclear magnetic resonance (NMR) [67], microimaging [34]) or indirect measurements of water dynamics under static conditions. In particular, indirect methods allow both to deduce *D* by measuring other physical properties of CNAs and to investigate effects of water confinement on electromagnetic, thermal, or optical water properties. For example, enhancement of *r*_1,2_ relaxivities of contrast agents for magnetic resonance imaging (MRI) is inversely proportional to *D*[68,69]; boiling temperature and pressure of water inside CNTs is drastically dependent on their diameter [70]; optical Kerr effect measurements are used to study modified characteristics and structures of nanoconfined water [71].

## Implications of the hypothesis

Molecular dynamics simulations and theoretical arguments suggest that self-diffusion of water *D* within CNAs can be finely tuned, from bulk to totally confined behaviors. As from Equation 8, two parameters control *D* in CNTs, namely *δ* characteristic length of nanoconfinement and *ϕ* nanotube diameter. Different *ϕ* distributions in CNAs can be experimentally achieved, according to a specific pattern, surface density, or width of the pores, in order to rationally design spatial *D* distribution of water. Nevertheless, while *ϕ* allows an *a priori* tuning of *D* (i.e., *D* distribution in CNAs cannot be modified once the sample is produced), the *a posteriori* control of *D* is feasible thanks to instantaneous modifications of *δ* by on/off switching or tuning of an external electrostatic field.

A broad range of CNT-based technologies may find benefits by the precise control of static transport properties of confined water molecules, in particular in biomedical or engineering fields. For example, MRI contrast agents performances are tuned by *D* of nearby water molecules; whereas the delivery rate of solvated drugs encapsulated within transdermal porous materials could be also controlled by *D* of surrounding water, both in constant release applications (e.g., long-term administration of therapeutics) and in dynamic ones (e.g., drug release in response to sudden pathological conditions). Moreover, the value of *D* in water is a key parameter in molecular sensors, sieves, and biological/chemical reaction chambers.

**Figure 5:**
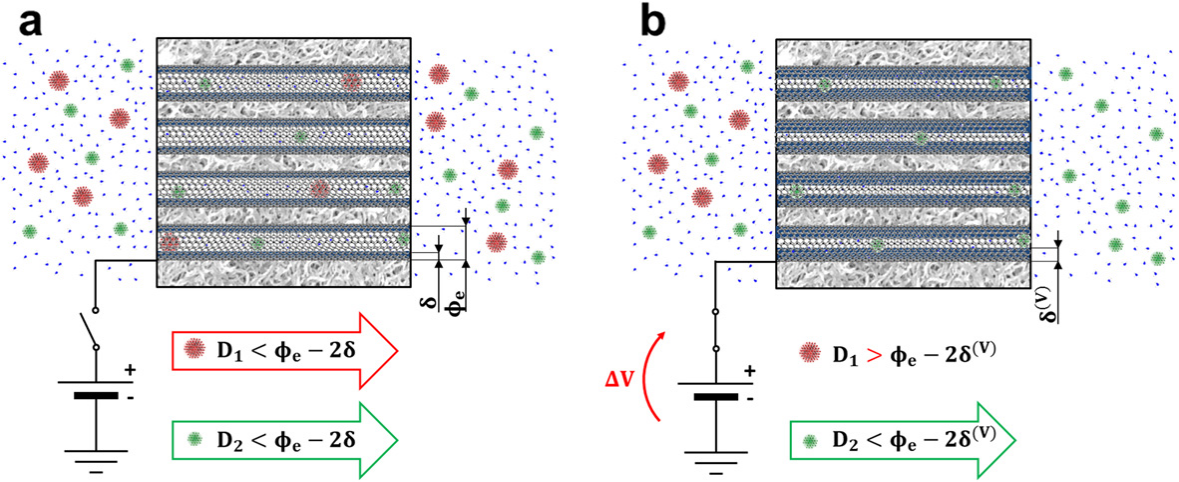
**Schematics of a tunable/switchable molecular sieve based on CNA. (a)** CNA-based composites may be used for sieving purposes, because their tunable pore width distribution induces different mass transport properties within pores. Thanks to Equations 4 and 7, CNA-based sieves may be designed for being selectively permeable to different nanoparticles or molecules, according to their size and chemical functionalization. **(b)** Sieving properties may also be influenced by the application of electrostatic field on CNA, which enhances *δ* of CNTs thus slowing down water dynamics and overall mass transport properties of the sieve. Hence, permeation features of CNAs may be precisely tuned and dynamically switched.

As an example, in Figure 5, we report the functional scheme of a possible molecular sieve based on *a posteriori* control (by electrostatic field) of *D* within a CNA-based composite. CNA should be adequately engineered with a *ϕ* size distribution so that only nanoparticles/molecules with approximately *D*<*ϕ*_*e*_-2*δ* diameters could pass through its nanopores (red and green spheres in Figure 5a). Note that chemical functionalization of both nanoparticles and CNTs may also influence the permeability of molecular sieve. Once an electrostatic field is applied to the CNA thus *δ* increases to *δ*^(*V*)^, sieving properties could suddenly change, due to reduced mobility of water within CNAs. For example, bigger nanoparticles/molecules could be trapped in the porous matrix (red spheres in Figure 5b), which could become partially or totally impermeable to nanoparticles/molecules with approximately *D*>*ϕ*_*e*_-2*δ*^(*V*)^ diameters. In fact, if CNA membranes are designed with well-defined pore sizes such that Barrer’s approximation holds and self- and transport diffusivities can be correlated by Darken’s equation (Equation 3), the increase in low-mobility water volume within CNTs may in turn affect the dynamic transport properties of CNA.

In this letter, self-diffusion of water within carbon nanotube arrays is studied, both numerically and theoretically. *D* is found to scale with CNT diameter *ϕ* and characteristic length of nanoconfinement *δ*, which is related to the solid-liquid nonbonded interaction potential at the interface. A strategy for an *a priori* modulation of *D* in CNA-based applications is suggested, by controlling size distribution of the pore widths. Moreover, *δ* of CNTs can be increased by introducing electrostatic field thus allowing also an *a posteriori* control of *D* in CNAs. A large variety of fields may benefit from a precise control of transport properties of water entrapped within CNA-based technologies, such as nanomedicine and environmental or energy engineering.

## Competing interests

The authors declare that they have no competing interests.

## Authors’ contributions

MF carried out the molecular dynamics simulations, proposed the idea of switchable molecular sieve, and drafted the manuscript. EC and PA conceived the idea and supervised the study. All authors refined and approved the final manuscript.

## References

[B1] Dresselhaus M Dresselhaus G Charlier J-C Hernandez E **Electronic, thermal and mechanical properties of carbon nanotubes** Philos Trans R Soc Lond A: Math Phys Eng Sci 2004 362 1823 2065 2098 10.1098/rsta.2004.1430 15370472

[B2] Chou T-W Gao L Thostenson ET Zhang Z Byun J-H **An assessment of the science and technology of carbon nanotube-based fibers and composites** Composites Sci Technol 2010 70 1 1 19 10.1016/j.compscitech.2009.10.004

[B3] Chiavazzo E Asinari P **Reconstruction and modeling of 3D percolation networks of carbon fillers in a polymer matrix** Intl J Thermal Sci 2010 49 12 2272 2281 10.1016/j.ijthermalsci.2010.07.019

[B4] Fasano M Bigdeli MB Sereshk Vaziri MR Chiavazzo E Asinari P **Thermal transmittance of carbon nanotube networks: guidelines for novel thermal storage systems and polymeric material of thermal interest** Renewable Sustainable Energy Rev 2015 41 1028 1036

[B5] Liu H Shen G **Ordered arrays of carbon nanotubes: from synthesis to applications** Nano Biomed Eng 2012 4 3 107 117

[B6] Gong J Sun L Zhong Y Ma C Li L Xie S Svrcek V **Fabrication of multi-level carbon nanotube arrays with adjustable patterns** Nanoscale 2012 4 1 278 283 10.1039/c1nr11191d 22080290

[B7] Jian S-R Chen Y-T Wang C-F Wen H-C Chiu W-M Yang C-S **The influences of H2 plasma pretreatment on the growth of vertically aligned carbon nanotubes by microwave plasma chemical vapor deposition** Nanoscale Res Lett 2008 3 6 230 235 10.1007/s11671-008-9141-5

[B8] Miyako E Sugino T Okazaki T Bianco A Yudasaka M Iijima S **Self-assembled carbon nanotube honeycomb networks using a butterfly wing template as a multifunctional nanobiohybrid** ACS nano 2013 7 10 8736 8742 10.1021/nn403083v 23952240

[B9] Roxbury D Jagota A Mittal J **Structural characteristics of oligomeric dna strands adsorbed onto single-walled carbon nanotubes** J Phys Chem B 2012 117 1 132 140 23199189 10.1021/jp309523a

[B10] Hinds BJ Chopra N Rantell T Andrews R Gavalas V Bachas LG **Aligned multiwalled carbon nanotube membranes** Science 2004 303 5654 62 65 10.1126/science.1092048 14645855

[B11] Holt JK Park HG Wang Y Stadermann M Artyukhin AB Grigoropoulos CP Noy A Bakajin O **Fast mass transport through sub-2-nanometer carbon nanotubes** Science 2006 312 5776 1034 1037 10.1126/science.1126298 16709781

[B12] Majumder M Chopra N Hinds BJ **Mass transport through carbon nanotube membranes in three different regimes: ionic diffusion and gas and liquid flow** ACS nano 2011 5 5 3867 3877 10.1021/nn200222g 21500837

[B13] Hinds B **Dramatic transport properties of carbon nanotube membranes for a robust protein channel mimetic platform** Curr Opin Solid State Mater Sci 2012 16 1 1 9 10.1016/j.cossms.2011.05.003

[B14] **Designing biomimetic pores based on carbon nanotubes** Proc Natl Acad Sci 2012 109 18 6939 6944 10.1073/pnas.1119326109 22509000 PMC3344987

[B15] Wu J Paudel KS Strasinger C Hammell D Stinchcomb AL Hinds BJ **Programmable transdermal drug delivery of nicotine using carbon nanotube membranes** Proc Natl Acad Sci 2010 107 26 11698 11702 10.1073/pnas.1004714107 20547880 PMC2900688

[B16] Santiago-Rodríguez L Sánchez-Pomales G Cabrera CR **Electrochemical dna sensing at single-walled carbon nanotubes chemically assembled on gold surfaces** Electroanalysis 2010 22 23 2817 2824 10.1002/elan.201000305

[B17] Yeh I-C Hummer G **Nucleic acid transport through carbon nanotube membranes** Proc Natl Acad Sci of the United States of America 2004 101 33 12177 12182 10.1073/pnas.0402699101 PMC514453 15302940

[B18] Liu H He J Tang J Liu H Pang P Cao D Krstic P Joseph S Lindsay S Nuckolls C **Translocation of single-stranded dna through single-walled carbon nanotubes** Science 2010 327 5961 64 67 10.1126/science.1181799 20044570 PMC2801077

[B19] Lulevich V Kim S Grigoropoulos CP Noy A **Frictionless sliding of single-stranded dna in a carbon nanotube pore observed by single molecule force spectroscopy** Nano Lett 2011 11 3 1171 1176 10.1021/nl104116s 21275410

[B20] Ramallo MV **An effective-charge model for the trapping of impurities of fluids in channels with nanostructured walls** Nanoscale Res Lett 2013 8 1 1 7 10.1186/1556-276X-8-1 23302600 PMC3600046

[B21] Anastassiou A Karahaliou EK Alexiadis O Mavrantzas VG **Detailed atomistic simulation of the nano-sorption and nano-diffusivity of water, tyrosol, vanillic acid, and p-coumaric acid in single wall carbon nanotubes** J Chem Phys 2013 139 16 164711 10.1063/1.4825397 24182068

[B22] Humplik T Lee J O’Hern S Fellman B Baig M Hassan S Atieh M Rahman F Laoui T Karnik R Wang E **Nanostructured materials for water desalination** Nanotechnology 2011 22 29 292001 10.1088/0957-4484/22/29/292001 21680966

[B23] Yang HY Han ZJ Yu SF Pey KL Ostrikov K Karnik R **Carbon nanotube membranes with ultrahigh specific adsorption capacity for water desalination and purification** Nat Commun 2013 4 2220 23941894 10.1038/ncomms3220

[B24] Gethard K Sae-Khow O Mitra S **Water desalination using carbon-nanotube-enhanced membrane distillation** ACS Appl Mater Interfaces 2010 3 2 110 114 21188976 10.1021/am100981s

[B25] Yoon D Lee C Yun J Jeon W Cha BJ Baik S **Enhanced condensation, agglomeration, and rejection of water vapor by superhydrophobic aligned multiwalled carbon nanotube membranes** ACS Nano 2012 6 7 5980 5987 10.1021/nn3008756 22732327

[B26] Zhou J Liu H Wang F Simpson T Sham T-K Sun X Ding Z **An electrochemical approach to fabricating honeycomb assemblies from multiwall carbon nanotubes** Carbon 2013 59 0 130 139

[B27] Li S Li H Wang X Song Y Liu Y Jiang L Zhu D **Super-hydrophobicity of large-area honeycomb-like aligned carbon nanotubes** J Phys Chem B 2002 106 36 9274 9276 10.1021/jp0209401

[B28] He S Wei J Wang H Sun D Yao Z Fu C Xu R Jia Y Zhu H Wang K He S Wei J Wang H Sun D Yao Z Fu C Xu R Jia Y Zhu H Wang K Wu D **Stable superhydrophobic surface of hierarchical carbon nanotubes on Si micropillar arrays** Nanoscale Res Lett 2013 8 1 1 6 10.1186/1556-276X-8-1 24098965 PMC3874759

[B29] Dhiman P Yavari F Mi X Gullapalli H Shi Y Ajayan PM Koratkar N **Harvesting energy from water flow over graphene** Nano Lett 2011 11 8 3123 3127 10.1021/nl2011559 21749100

[B30] Persson B Tartaglino U Tosatti E Ueba H **Electronic friction and liquid-flow-induced voltage in nanotubes** Phys Rev B 2004 69 23 235410

[B31] Zhao G Bagayoko D Yang L **Optical properties of aligned carbon nanotube mats for photonic applications** J Appl Phys 2006 99 11 114311 114311 10.1063/1.2201738

[B32] Marconnet AM Panzer MA Goodson KE **Thermal conduction phenomena in carbon nanotubes and related nanostructured materials** Rev Modern Phys 2013 85 3 1295 10.1103/RevModPhys.85.1295

[B33] Nanok T Artrith N Pantu P Bopp PA Limtrakul J **Structure and dynamics of water confined in single-wall nanotubes** J Phys Chem A 2008 113 10 2103 2108 19115825 10.1021/jp8088676

[B34] Kärger J Binder T Chmelik C Hibbe F Krautscheid H Krishna R Weitkamp J **Microimaging of transient guest profiles to monitor mass transfer in nanoporous materials** Nat Mater 2014 13 4 333 343 10.1038/nmat3917 24651427

[B35] Du F Qu L Xia Z Feng L Dai L **Membranes of vertically aligned superlong carbon nanotubes** Langmuir 2011 27 13 8437 8443 10.1021/la200995r 21657212

[B36] Qin X Yuan Q Zhao Y Xie S Liu Z **Measurement of the rate of water translocation through carbon nanotubes** Nano Lett 2011 11 5 2173 2177 10.1021/nl200843g 21462938

[B37] Melillo M Zhu F Snyder MA Mittal J **Water transport through nanotubes with varying interaction strength between tube wall and water** J Phys Chem Lett 2011 2 23 2978 2983 10.1021/jz2012319 PMC4297660 25606067

[B38] Chiavazzo E Fasano M Asinari P Decuzzi P **Scaling behaviour for the water transport in nanoconfined geometries** Nat Commun 2014 5 4495 24699509 10.1038/ncomms4565 PMC3988813

[B39] Liu Y Wang Q Wu T Zhang L **Fluid structure and transport properties of water inside carbon nanotubes** J Chem Phys 2005 123 23 234701 10.1063/1.2131070 16392938

[B40] Taylor R Krishna R Multicomponent Mass Transfer 1993 New York: Wiley

[B41] Beerdsen E Dubbeldam D Smit B **Understanding diffusion in nanoporous materials** Phys Review Lett 2006 96 4 044501 10.1103/PhysRevLett.96.044501 16486827

[B42] Krishna R **Describing the diffusion of guest molecules inside porous structures** J Phys Chem C 2009 113 46 19756 19781 10.1021/jp906879d

[B43] Krishna R **Diffusion in porous crystalline materials** Chem Soc Rev 2012 41 8 3099 3118 10.1039/c2cs15284c 22262346

[B44] Krishna R van Baten JM **Influence of adsorption thermodynamics on guest diffusivities in nanoporous crystalline materials** Phys Chem Chem Phys 2013 15 21 7994 8016 10.1039/c3cp50449b 23628965

[B45] Asinari P **Semi-implicit-linearized multiple-relaxation-time formulation of lattice Boltzmann schemes for mixture modeling** Phys Rev E 2006 73 5 056705 10.1103/PhysRevE.73.056705 16803072

[B46] Kjelstrup S Bedeaux D Non-equilibrium Thermodynamics of Heterogeneous Systems 2008 Singapore: World Scientific

[B47] Barrer R Jost W **A note on interstitial diffusion** Trans Faraday Soc 1949 45 928 930

[B48] Barrer RM Zeolites and Clay Minerals as Sorbents and Molecular Sieves 1978 Waltham: Academic Press

[B49] Chmelik C Bux H Caro J Heinke L Hibbe F Titze T Karger J **Mass transfer in a nanoscale material enhanced by an opposing flux** Phys Rev Lett 2010 104 8 085902 20366950 10.1103/PhysRevLett.104.085902

[B50] Gallo P Rovere M Spohr E **Supercooled confined water and the mode coupling crossover temperature** Phys Rev Lett 2000 85 20 4317 10.1103/PhysRevLett.85.4317 11060627

[B51] Chen S-H Mallamace F Mou C-Y Broccio M Corsaro C Faraone A Liu L **The violation of the Stokes–Einstein relation in supercooled water** Proc Natl Acad Sci 2006 103 35 12974 12978 10.1073/pnas.0603253103 16920792 PMC1559737

[B52] Quo Y Karasawa N Goddard WA **Prediction of fullerene packing in c60 and c70 crystals** Nature 1991 351 464 467 10.1038/351464a0

[B53] Eisenhaber F Lijnzaad P Argos P Sander C Scharf M **The double cubic lattice method: efficient approaches to numerical integration of surface area and volume and to dot surface contouring of molecular assemblies** J Comput Chem 1995 16 3 273 284 10.1002/jcc.540160303

[B54] Berendsen H Grigera J Straatsma T **The missing term in effective pair potentials** J Phys Chem 1987 91 24 6269 6271 10.1021/j100308a038

[B55] Bussi G Donadio D Parrinello M **Canonical sampling through velocity rescaling** J Chem Phys 2007 126 1 014101 10.1063/1.2408420 17212484

[B56] Chiavazzo E Asinari P **Enhancing surface heat transfer by carbon nanofins: towards an alternative to nanofluids?** Nanoscale Res Lett 2011 6 1 1 13 10.1186/1556-276X-6-249 PMC3211310 21711780

[B57] Walther JH Jaffe R Halicioglu T Koumoutsakos P **Carbon nanotubes in water: structural characteristics and energetics** J Phys Chem B 2001 105 41 9980 9987 10.1021/jp011344u

[B58] Hess B Kutzner C Van Der Spoel D Lindahl E **Gromacs 4: algorithms for highly efficient, load-balanced, and scalable molecular simulation** J Chem Theory Comput 2008 4 3 435 447 10.1021/ct700301q 26620784

[B59] van der Spoel D **A systematic study of water models for molecular simulation: derivation of water models optimized for use with a reaction field** J Chem Phys 1998 108 24 10220 10230 10.1063/1.476482

[B60] Ge L Wang L Du A Hou M Rudolph V Zhu Z **Vertically-aligned carbon nanotube membranes for hydrogen separation** RSC Advances 2012 2 12 5329 5336 10.1039/c2ra00031h

[B61] He M Vasala S Jiang H Karppinen M Kauppinen EI Niemelä M Lehtonen J **Growth and surface engineering of vertically-aligned low-wall-number carbon nanotubes** Carbon 2012 50 12 4750 4754 10.1016/j.carbon.2012.05.028

[B62] Sears K Dumée L Schütz J She M Huynh C Hawkins S Duke M Gray S **Recent developments in carbon nanotube membranes for water purification and gas separation** Materials 2010 3 1 127 149 10.3390/ma3010127

[B63] Cazade P-A Hartkamp R Coasne B **Structure and dynamics of an electrolyte confined in charged nanopores** J Phys Chem C 2014 118 10 5061 5072 10.1021/jp4098638

[B64] Frackowiak E Jurewicz K Delpeux S Beguin F **Nanotubular materials for supercapacitors** J Power Sources 2001 97 822 825

[B65] Pan H Li J Feng YP **Carbon nanotubes for supercapacitor** Nanoscale Res Lett 2010 5 3 654 668 10.1007/s11671-009-9508-2 20672061 PMC2894167

[B66] Frackowiak E Khomenko V Jurewicz K Lota K Beguin F **Supercapacitors based on conducting polymers/nanotubes composites** J Power Sources 2006 153 2 413 418 10.1016/j.jpowsour.2005.05.030

[B67] Cohen Y Avram L Frish L **Diffusion NMR spectroscopy in supramolecular and combinatorial chemistry: an old parameter–new insights** Angewandte Chemie International Edition 2005 44 4 520 554 10.1002/anie.200300637 15625667

[B68] Ananta JS Godin B Sethi R Moriggi L Liu X Serda RE Krishnamurthy R Muthupillai R Bolskar RD Helm L Ananta JS Godin B Sethi R Moriggi L Liu X Serda RE Krishnamurthy R Muthupillai R Bolskar RD Helm L Ferrari M Wilson LJ Decuzzi P **Geometrical confinement of gadolinium-based contrast agents in nanoporous particles enhances T1 contrast** Nat Nanotechnol 2010 5 11 815 821 10.1038/nnano.2010.203 20972435 PMC2974055

[B69] Gizzatov A Key J Aryal S Ananta J Cervadoro A Palange AL Fasano M Stigliano C Zhong M Di Mascolo D Guven A Chiavazzo E Asinari P Liu X Ferrari M Wilson LJ Decuzzi P **Hierarchically structured magnetic nanoconstructs with enhanced relaxivity and cooperative tumor accumulation** Adv Funct Mater 2014 24 29 4584 4594 10.1002/adfm.201400653 PMC4497786 26167143

[B70] Chaban VV Prezhdo OV **Water boiling inside carbon nanotubes: toward efficient drug release** ACS Nano 2011 5 7 5647 5655 10.1021/nn201277a 21648482

[B71] Taschin A Bartolini P Marcelli A Righini R Torre R **A comparative study on bulk and nanoconfined water by time-resolved optical kerr effect spectroscopy** Faraday Discuss 2013 167 293 308 24640497 10.1039/c3fd00060e

